# Supplementary material for: Determination of Antioxidant, Cytotoxicity, and Acetylcholinesterase Inhibitory Activities of Alkaloids Isolated from Sophora flavescens Ait. Grown in Dak Nong, Vietnam
Source: Pharmaceuticals (Basel). 2022 Nov 10;15(11):1384. doi: 10.3390/ph15111384 (PMC9696406; doi:10.3390/ph15111384)
Supplement: Supplementary file 1 [file pharmaceuticals-15-01384-s001.zip › pharmaceuticals-1990532-supplementary.pdf]

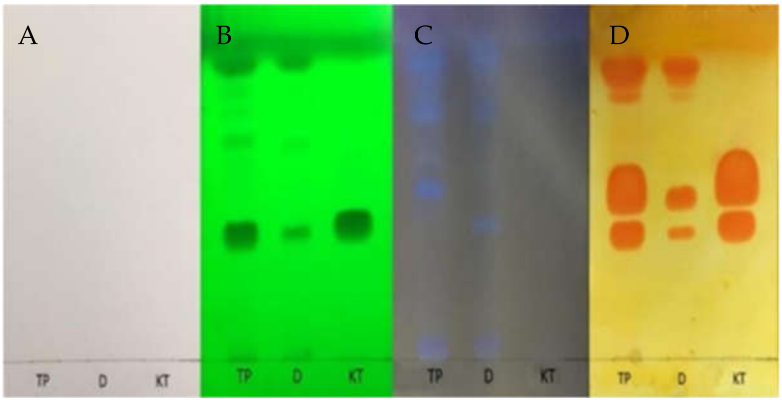

Figure S1. Thin-layer chromatography of the *Sophora flavescens* Ait. chloroform fraction under (**A**) visible light, (**B**) UV 254 nm, (**C**) UV 365 nm, and (**D**) Dragendorff reagent. TP: chloroform total extract; D: non-crystallized portion; KT: crystalline portion.

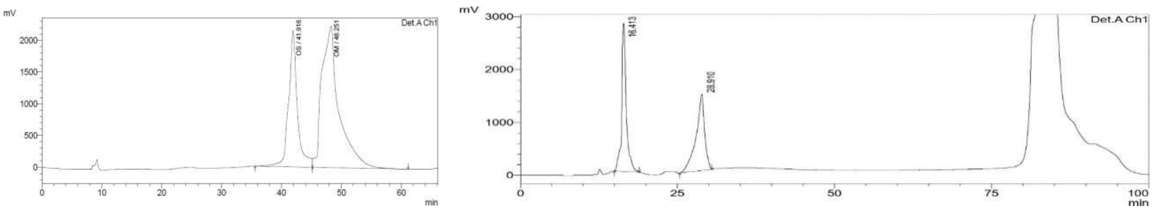

Figure S2. HPLC chromatograms of the *Sophora flavescens* Ait. chloroform fraction. (**Left**) The crystalline portion and (**Right**) the non-crystallized portion.

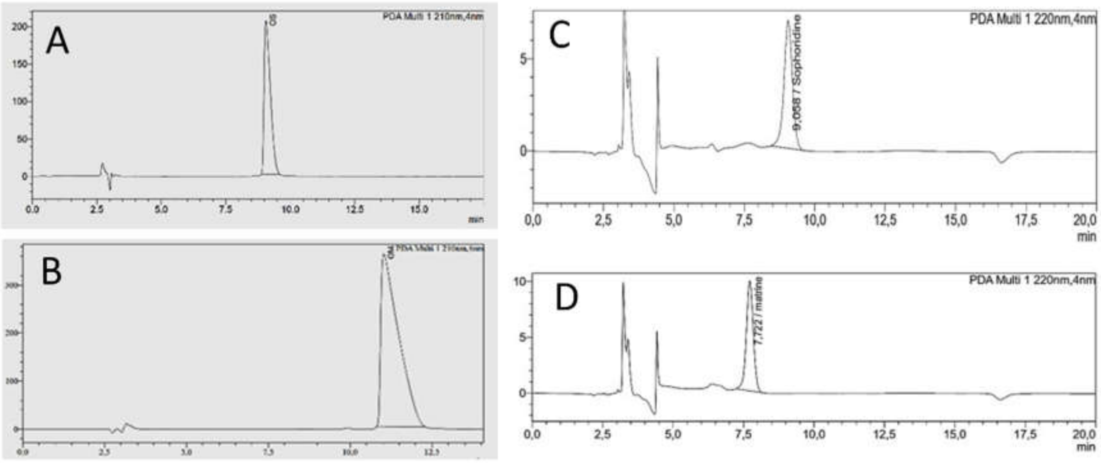

Figure S3. The purity, confirmed by the HPLC analysis, of the 04 compounds of (**A**) A1, (**B**) A2, (**C**) A3, and (**D**) A4, isolated from the *Sophora flavescens* Ait. root.
